# Academics and competing interests in H1N1 influenza media reporting

**DOI:** 10.1136/jech-2013-203128

**Published:** 2013-11-11

**Authors:** Kate L Mandeville, Sam O'Neill, Andrew Brighouse, Alice Walker, Kielan Yarrow, Kenneth Chan

**Affiliations:** 1 Department of Global Health and Development, London School of Hygiene and Tropical Medicine, London, UK; 2 School of Medicine, Imperial College London, London, UK; 3 Accident and Emergency Department, Princess Alexandra Hospital NHS Trust, Harlow, UK; 4 Accident and Emergency Department, Chase Farm Hospital, Enfield, UK; 5 Department of Psychology, City University London, London, UK; 6 Barts and The London School of Medicine & Dentistry, London, UK

**Keywords:** PUBLIC HEALTH POLICY, INFLUENZA, ETHICS, EPIDEMICS

## Abstract

**Background:**

Concerns have been raised over competing interests (CoI) among academics during the 2009 to 2010 A/H1N1 pandemic. Media reporting can influence public anxiety and demand for pharmaceutical products. We assessed CoI of academics providing media commentary during the early stages of the pandemic.

**Methods:**

We performed a retrospective content analysis of UK newspaper articles on A/H1N1 influenza, examining quoted sources. We noted when academics made a risk assessment of the pandemic and compared this with official estimations. We also looked for promotion or rejection of the use of neuraminidase inhibitors or H1N1-specific vaccine. We independently searched for CoI for each academic.

**Results:**

Academics were the second most frequently quoted source after Ministers of Health. Where both academics and official agencies estimated the risk of H1N1, one in two academics assessed the risk as higher than official predictions. For academics with CoI, the odds of a higher risk assessment were 5.8 times greater than those made by academics without CoI (Wald p value=0.009). One in two academics commenting on the use of neuraminidase inhibitors or vaccine had CoI. The odds of CoI in academics promoting the use of neuraminidase inhibitors were 8.4 times greater than for academics not commenting on their use (Fisher's exact p=0.005).

**Conclusions:**

There is evidence of CoI among academics providing media commentary during the early H1N1 pandemic. Heightened risk assessments, combined with advocacy for pharmaceutical products to counter this risk, may lead to increased public anxiety and demand. Academics should declare, and journalists report, relevant CoI for media interviews.

## Introduction

The UK spent an estimated one billion pounds on pharmaceutical products during the 2009 to 2010 A/H1N1 influenza pandemic, including neuraminidase inhibitors (NI) and H1N1-specific vaccine.^1^ Pharmaceutical companies made profits of 4.5–6.5 billion pounds from H1N1 vaccines alone.^2^ This was despite the evaluation of the pandemic as less severe than previous pandemics^3^ ^4^ and uncertainty over the effectiveness of neuraminidase inhibitors (a type of antiviral medication) in reducing transmission and complications of influenza.^5^

In the postpandemic period, there were significant concerns about competing interests (CoI) among experts on influential advisory committees, including the WHO Emergency Committee.^2^ ^6^ ^7^ Members of these committees have been linked to manufacturers of both neuraminidase inhibitors and influenza vaccines.^7^ ^8^ There have been repeated calls for greater transparency around the potential influence of the pharmaceutical industry on the decisions made by these committees.^2^ ^6^ ^7^ ^9^

Public health academics are often asked to provide commentary and analysis on emerging health risks by the media. Media coverage of health issues has been shown to influence the public's perception of risk, demand for new drugs and policy decisions.^10–13^ In the UK, extensive media advocacy of the breast cancer drug trastuzumab (Herceptin) resulted in a ‘fast-track’ approval from the National Institute for Health and Clinical Excellence,^14^ but there was subsequent debate over the cost-effectiveness of the drug.^15^ It has been suggested that optimistic media portrayals may be more successful for pharmaceutical companies than explicit promotional campaigns as “the message is separated from an obvious marketing agenda and often includes a trusted voice, such as a university-based researcher. Paradoxically, this trust is based in part on a belief in the perceived independence of university researchers”.^16^ Like those on advisory committees, academics quoted in the media may also have possible CoI. Media commentaries, therefore, represent an alternative route to exert pressure on public demand and one in which CoI are not routinely declared.

We set out to examine media commentary on A/H1N1 influenza provided by academics during the period in which the UK government decided its policy on public provision of NI and H1N1-specific vaccine (NI/vaccine). We then independently searched for CoI for each academic to determine whether commentary from academics with and without CoI was significantly different.

## Methods

### Design and setting

This study was a retrospective content analysis of UK newspaper reporting. We excluded television and radio coverage, as audiovisual reporting is often limited by time constraints and presents less divergent viewpoints and in-depth analysis compared with print media.^17^ ^18^

### Selection of newspaper articles

**Figure 1:**
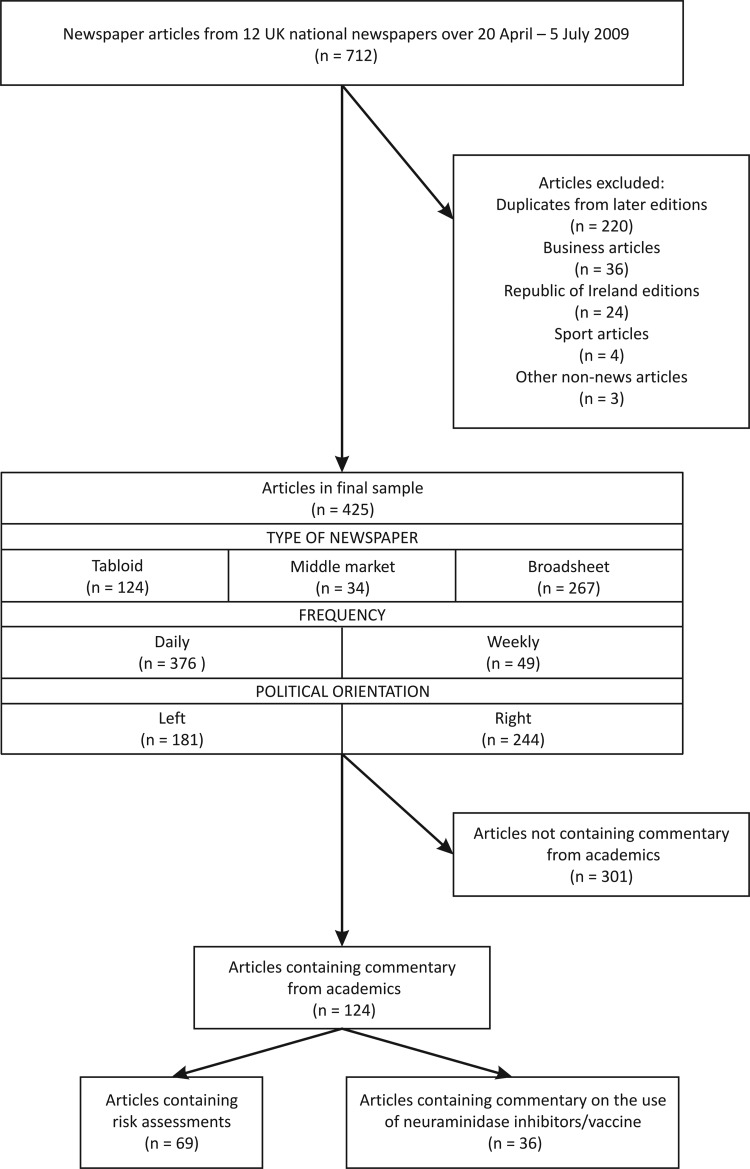
Flow of articles through study.

Figure 1 shows the flow of articles through the study. We used the Nexis-UK database, which provides full-text access to all UK national newspapers. Twelve UK national newspapers were included in the sample (January 2009 circulation figures are given in parentheses^19^): *Daily Mirror* (1 366 891), *Sunday Mirror* (1 244 007), *The Sun* (3 146 006), *News of the World* (3 031 025), *Daily Mail* (2 200 398), *The Mail on Sunday* (2 134 809), *The Guardian* (358 844), *The Independent* (215 504), *The Observer* (427 867), *Daily Telegraph* (783 210), *The Times* (617 483) and *The Sunday Times* (1 198 984). These were selected in order to ensure coverage from tabloid, middle-market and broadsheet publications, daily and Sunday newspapers, and left and right political orientations so that a range of perspectives and reporting styles were represented. This typology has been used in previous content analyses.^20^ ^21^

The database was searched using the following terms (an exclamation mark is used as a truncator in this database): H1N1, Influenza A, Swine !flu!, Pandemic !flu!, Pig !flu!. Only articles that contained at least three mentions of the search terms were eligible for inclusion in order to select articles where H1N1 influenza was the main theme. Articles with a different focus entirely, such as business, sports and non-news articles like obituaries, were excluded. Search dates were between 20 April and 5 July 2009, the period in which the major decisions on pharmaceuticals as part of the pandemic response were taken by the UK government. Key events and policy decisions within this period are summarised in table 1.^1^ ^22^ News coverage dropped off considerably after this period.^20^

**Table 1:** Key events, official risk assessments and UK policy decisions during study period

Date (2009)	Event/policy decision
Week of 20 April	First human cases of H1N1 confirmed in Mexico, the USA and Canada.
24 April	HPA press release: “The mild illness reported to date and the limited evidence of sustained human-to-human transmission suggest that the immediate level of threat to public health is very limited”.
26 April	UK government agrees to containment measures as part of its emergency response, including treatment of suspected cases and their close contacts with neuraminidase inhibitors without waiting for diagnostic confirmation.
27 April	Confirmation of first UK cases. Minister of Health issues statement: “The range of symptoms in the people affected is similar to those of regular human seasonal influenza. It is important to note that, apart from in Mexico, all those infected with the virus have experienced mild symptoms and made a full recovery”.
29 April	WHO states, “It is possible that the full clinical spectrum of this disease goes from mild illness to severe disease. We need to continue to monitor the evolution of the situation...”. UK government decides to increase the national stockpile of neuraminidase inhibitors from 33.5 million to 50 million doses.
1 May	HPA confirms human-to-human transmission in UK, stating: “At this stage, we still only have two cases of human to human transmission in the UK. This does not yet represent sustained human to human transmission. The risk to the general public is still very low”.
11 May	UK government takes decision to purchase sufficient H1N1-specific vaccine for 45% of the population.
11 June	WHO confirms start of a global pandemic, stating “we have good reason to believe that this pandemic, at least in its early days, will be of moderate severity. Worldwide, the number of deaths is small. [..]..we do not expect to see a sudden and dramatic jump in the number of severe or fatal infections”.
15 June	DH statement: “The localised cases of swine flu found in the UK have so far been generally mild in most people, but are proving to be severe in a small minority of cases”.
17 June	WHO welcomes donation by Sanofi-Aventis of 100 million doses of H1N1 vaccine for low-income countries.
26 June	GlaxoSmithKline and Baxter Healthcare contracted to provide a total of 132 million doses of H1N1-specific vaccine, sufficient for two doses for the whole UK population.
2 July	UK government changes to ‘treatment’ phase in its emergency response, where prophylaxis with neuraminidase inhibitors would be provided to those in high-risk groups only. HPA press release states: “Once the virus is widespread within the community, the value of antivirals in terms of slowing the spread of the disease or offering individual protection is greatly reduced”.

DH, Department of Health (England); HPA, Health Protection Agency.

Using these criteria, 712 articles were eligible for inclusion. These were extracted into Microsoft Word and screened by one of the authors. Duplicate articles from later editions of the newspapers and any remaining articles as per exclusion criteria above were excluded, leaving 425 articles in the final sample. These provided a good coverage of formats, frequencies and political orientation, taking into account the circulation figures above (figure 1).

### Coding framework

Each article was assessed independently by two authors using a standardised coding framework consisting of two sections.

The first section categorised the sources quoted in each article. The main categories consisted of Health Secretary/Minister (of England and the Devolved Administrations—Wales, Scotland and Northern Ireland); Department of Health (of England and the Devolved Administrations); Chief Medical Officer (of England and the Devolved Administrations); World Health Organization (WHO); the UK's Health Protection Agency (HPA), the Centers for Disease Prevention and Control (CDC); pharmaceutical company representative; and named academic (defined here as a researcher or academic clinician affiliated with a higher educational body or research institute in the article).

The second section looked in greater detail at those articles that quoted academic sources. First, we examined whether academics made a risk assessment of the emerging pandemic. For example, quotes such as “this is going to affect millions of people in England” or “thousands of people could die from this virus” would be a risk assessment. We then checked whether the academic cited official figures or whether there was a risk assessment made by an official body relevant to the UK population quoted within the same article (defined as WHO, Health Secretary/Minister(s), Chief Medical Officer(s), Department(s) of Health or HPA). Table 1 presents examples of risk assessments from these agencies during the study period. We used the official risk assessments as a benchmark to measure each academic risk assessment: judging whether it concurred with the official estimate, or was higher or lower (ie, implying more or less risk to the public).

All quotes by academics were then examined for reference to the use of NI or influenza vaccine. Those that made reference to NI/vaccine were further analysed as to whether they promoted or rejected the use of these products. The analysis was performed according to a pre-agreed consensus on terms. ‘Promotion’ was defined as advocacy of the effectiveness, need for or supply shortages of NI/vaccine. Conversely, ‘rejection’ referred to statements highlighting the adverse effects, ineffectiveness of or lack of need for NI/vaccine.

The coding framework was piloted on 20 articles by both coders, with subsequent minor modifications made to definitions before coding of the complete data set. Cohen's kappa was calculated to determine inter-rater agreement between the qualitative measures of risk assessment and promotion/rejection of pharmaceutical products.^23^ ^24^ Disagreements between coders were assessed by a third researcher for final arbitration. Microsoft Excel was used for all coding and calculations.

### Evidence of CoI

For each named academic, we performed a comprehensive search for CoI based on the protocol from a recent study examining CoI in authors of clinical practice guidelines.^25^ This was undertaken by two researchers who did not take part in the coding in order to minimise bias. We used the International Committee of Medical Journal Editors’ definition that “Conflicts of interests exists when an author (…) has financial or personal relationships that inappropriately influence (bias) his or her actions)”.^26^ For each academic, we looked for associations with pharmaceutical or biotechnology companies, in the form of grants (including research), honorariums, speakers’ fees, consultant/adviser/employee relationships and stock ownership.^25^ These could be personal, indicating benefit to that individual (eg, honorariums), or non-personal, indicating benefit to a department or organisation for which an academic has managerial responsibility (eg, research grants).^16^ We searched for CoI from the 4 years before the start of the pandemic, that is, March 2005 to March 2009. This is consistent with the WHO's standard that CoI should be declared if incurred in the 4 years before acting in an expert advisory role.^25^ ^27^

For each academic, we made the following searches in a sequential manner, stopping after each stage if a CoI was identified:

- The CoI statements (where available) for four major scientific advisory committees relevant to this issue: Joint Committee on Vaccination and Immunisation (UK), Scientific Advisory Group on Emergencies (UK), WHO Emergency Committee and WHO Strategic Advisory Group of Experts.
- Funding sources detailed on the individual's profile page on the website of affiliated institution.
- A general internet search using Google linking “(name of academic)” with respectively “vaccine”, “neuraminidase inhibitor”, “antiviral”, “Oseltamivir”, “Zanamivir” and the name of the main pharmaceutical companies producing neuraminidase inhibitors (Roche, GlaxoSmithKline) and influenza vaccine (Novartis, GlaxoSmithKline, Baxter International, Sanofi-Pasteur). The list of manufacturers was obtained through the electronic Medicines Compendium (http://www.emc.medicines.org.uk).
- CoI and funding declarations on all publications in the past 4 years. These were identified through the PubMed/Medline database.

Two authors identified CoI, and a separate author verified the presence of CoI.

We calculated the likelihood of a risk assessment being higher than official estimates if it was made by an academic with CoI compared with those without CoI. As some academics made multiple risk assessments, we used a variant of the generalised linear model (generalised estimating equations, using a binary logistic link function, with an exchange correlation matrix) to take account of clustering.^28^ We also calculated the likelihood of an academic who promoted or rejected the use of NI/vaccine having CoI compared with academics who provided general commentary, using Fisher's exact test. All statistics were calculated in SPSS V.19.

## Results

### Quoted sources

Ministers of Health were the most frequently quoted sources (144/425, 33.9% of articles), while academics were the second most commonly quoted (29.7%, 126/425). Other common sources included WHO (27.8%, 118/425), Departments of Health (21.6%, 92/425), HPA (19.1%, 81/425), Chief Medical Officers (16.2%, 69/425) and CDC (5.6%, 24/425). Pharmaceutical companies were quoted in just eight articles (1.9%). A total of 61 named academics were quoted within the sample.

### Risk assessments

Academics made 74 risk assessments, over half of which were higher than with those made by official agencies in the same article (59.5%, 44/74). In nearly a quarter, 23.0%, 17/74), academics concurred with official risk assessments and in 17.6% (13/74), academics estimated the risk as lower. Table 2 gives some examples of these different categories of risk assessments.

**Table 2:** Examples of risk assessments made by academics and official agencies, by category assigned to academic risk assessment

	Official risk assessment	Academic risk assessment
Higher than official agencies	“..between 400 000 and 800 000 people [become] ill in an average flu season, but [at the peak of a pandemic] you would probably be into several million cases” [Chief Medical Officer]	“The virus [is] likely to be two to three times more deadly than seasonal flu...the pandemic could mean that 25–35 per cent of the population would fall ill within three or four months, placing severe strain on the NHS”.
Concurring with official agencies	Minister of Health: “There is no cause for anyone to feel there is going to be any danger to them at this stage... Pandemics come along every 20 years and the present outbreak [is] not inevitably going to move to level six”, however [*the Minister of Health*] indicated that he thought it likely that the alert level might rise to pandemic.”	“We haven't yet identified any features that give us cause for concern, or that indicate high virulence [...]. It is important that people keep a sense of perspective, because what we observe is what may lead to a pandemic. We don't know that it will lead to a pandemic, although many of us think that this is highly likely”.
Lower than official agencies	“Even though the fatality rate is relatively low we will see a lot of people dying because of the large number of people being infected. As more and more cases are reported in the US, we are starting to see some hospitalisations and more severe cases. We may see the same pattern in the UK”. [*World Health Organization*]	“This might not be any more virulent than normal seasonal flu infections. We feel reassured that if this develops into a pandemic it might not be a particularly severe one”.

### Use of NI/vaccine

Twenty academics commented specifically on the use of NI/vaccine in 36 articles (8.5% of total articles). Ten academics (50%) promoted the use of NI whereas four specifically rejected their use (20%). Nine academics (45%) promoted the use of a vaccine, while none rejected its use. Three academics (15%) promoted the use of both NI and vaccine. Examples of quotes for these categories are illustrated in table 3. Cohen's kappa for inter-rater agreement was 0.66 (values between 0.61 and 0.80 indicate substantial inter-rater agreement).^24^

**Table 3:** Comments promoting or rejecting the use of neuraminidase inhibitors or vaccine

Type of comment	Example
Promoting the use of neuraminidase inhibitors	“There is no doubt Tamiflu [oseltamivir] will help”.
	“There is an issue of Tamiflu resistance. All things being equal, it would be nice to get as much Relenza [zanamivir] as we can get our hands on”.
Promoting the use of vaccine	“I think by far the safer option is to wait for the development of a vaccine which will almost certainly be around by the autumn”.
	“Vaccines are our real hope”.
Rejecting the use of neuraminidase inhibitors	“At present it [Tamiflu] should not be routinely prescribed”.
	“No one really knows if Tamiflu will significantly reduce transmission; the expectation is it will, but we don't know for sure”.

### Competing interests

A total of 61 named academics were quoted within the sample. We identified CoI in a third of these academics (29.5%, 18/61), through CoI declarations for scientific advisory committees (5), profile pages (2), internet searches (9) and publications (2). Most CoI were personal in nature (13/18, 72.2%), consisting of paid consultancies or advisory roles, directorships or stock in companies specialising in antiviral products. Seven CoI were non-personal (38.9%), relating to research grants or commercial work funded by pharmaceutical companies. Two academics held both personal and non-personal CoI.

Out of the 44 risk assessments that were higher than official sources, 35 were made by academics with CoI. In contrast, 10 of the 30 risk assessments that concurred with or were lower than official sources were made by academics with CoI. As several academics made more than one risk assessment, data were fitted using generalised equalising equations. In this analysis, risk assessments were categorised as either being higher than official estimates or concurring with/lower than the official position, forming a binary dependent variable. The best-fitting model revealed that for risk assessments made by academics with CoI the odds of a higher risk assessment were 5.8 times greater compared with assessments made by academics without CoI (Wald p value=0.009).

Out of the 20 academics who commented on the use of NI/vaccine in the pandemic, one in two had CoI (10, 50%). This is a higher proportion than the one in three academics on the WHO's Emergency Committee advisory group who declared CoI.^8^

When we correlated CoI by type of comment, 7 out of 10 academics (70%) promoting the use of NI had CoI compared with 10 out of 47 (21.3%) of academics not commenting on their use (table 4). The odds of COI in academics promoting the use of NI were 8.4 times greater than for academics not commenting on the use of NI (Fisher's exact p=0.005). The odds of CoI in academics rejecting the use of NI were not significantly different to the odds in those not commenting their use (Fisher's exact p=1.0). Five out of nine academics (55.6%) promoting the use of a vaccine had a CoI compared with 13 out of 52 (25.0%) not commenting on its use, a non-significant trend (Fisher's exact p=0.11).

**Table 4:** Number of academics with competing interests by type of comment

Type of comment	Number of academics	Number with competing interests (%)
Promoting the use of NI	10	7 (70)
Rejecting the use of NI	4	1 (25)
Not commenting on the use of NI	47	10 (21.3)
Promoting the use of vaccine	9	5 (55.6)
Rejecting the use of vaccine	0	0 (0)
Not commenting on the use of vaccine	52	13 (25.5)

NI, neuraminidase inhibitors.

Only three articles in the entire sample noted that the quoted academics had a potential conflict of interest, with one columnist commenting that, “it would be helpful if newspapers informed us of these things”.

## Discussion

During the period in which the UK government took its major decisions on pharmaceutical policy, one in two academics commenting on NI/vaccine use in UK national newspapers had CoI. The odds of CoI in academics promoting the use of NI were 8.4 times greater than for academics not commenting on the use of NI. If academics with CoI made an assessment of the risk of the pandemic, the odds of this risk assessment being higher than official sources were 5.8 times greater compared with assessments made by academics without CoI.

CoI among academics on influential advisory committees have led to intense debate worldwide.^2^ ^6^ ^7^ This study estimates, for the first time, the prevalence of CoI among academics providing media commentary during the early H1N1 pandemic. We combined a rigorous search for CoI with a comprehensive sample of nationally prominent media during a critical policymaking period. Our findings are based on a small sample, however, and should be viewed as a scoping study. They are corroborated by a study by Moynihan *et al*^29^ examining news coverage of three medications for non-communicable diseases, which found that out of 170 stories citing an expert or a scientific study, 50% (85) cited those with a financial tie to the drug manufacturer. Indeed, a study looking at UK newspapers’ representations of the H1N1 pandemic found little discussion of the profits that pharmaceutical companies would make from the development of a H1N1-specific vaccine and few articles describing the potential side effects of vaccines.^20^

It is clear from our results that academics constitute an accessible and trusted source for journalists. Academics were the second most commonly quoted source after Ministers of Health, and therefore hold a unique and powerful position for communication on emerging public health issues. However, in a third of cases, academics estimated the risk of the emerging pandemic as higher than official sources. We recognise that academics may be involved in modelling outcomes based on early estimates and may therefore predict higher risks than is borne out by more comprehensive data. In addition, journalists may seek out divergent viewpoints in order to provide balance within a story or to increase its newsworthiness. However, consensus among risk assessors during public health emergencies is important to decrease public anxiety, increase the effectiveness of risk communication and promote adherence to personal protective measures.^30–32^ We would suggest that this responsibility extends to the media as well, who may need to balance their investigative role with the need to provide a clear and consistent message during the early stages of a public health emergency. Indeed, content analyses of UK^20^ and European media reporting on H1N1 influenza^33^ found predominantly factual reporting with little evidence of sensationalism.

Our results provide some evidence that the provision of higher risk assessments and the promotion of NI are associated with CoI among academics. These add to the growing body of literature highlighting the potential influence of the pharmaceutical industry on policy decisions through multiple avenues, including advisory committees^6^, drafting of guidelines^25^ and media commentary.^16^ This type of influence may be stronger for more familiar health issues, such as cancer, as the public response to emerging health risks is usually one of scepticism.^30^ Indeed, uptake of H1N1-specific vaccine during the pandemic among those in clinical risk groups was only 37.6%,^34^ which suggests that both the official vaccination campaign and any media support for vaccination had limited impact.

There were several limitations to our study. Although this sample was drawn from a large number of articles, the number of academics actually commenting on the use of NI/vaccine was small. More quotes may have been obtained if the study period was extended to the end of the H1N1 pandemic in the UK, but any CoI would be less relevant after the main decisions on pharmaceutical products were taken. While newspaper articles provide a limited set of quotes, the actual interviews with academic sources were undoubtedly longer and may have contained more nuanced views than those represented by the quotes. The definitions and coding of promotion/rejection could be criticised as subjective, although similar definitions have been used in other content analyses.^35^ Finally, we performed a comprehensive search for CoI, but there may be further conflicts (disclosed or undisclosed) that were not identified here.

Rather than trying to decrease commentary on public health issues from academics with CoI, a pragmatic approach would be to focus on the complete transparency of these interests^36^ and allow readers in any capacity to judge comments from academics with these in mind. Indeed, there have been repeated calls for journalists to investigate CoI in their quoted sources in science articles.^16^ ^37^ ^38^ In the study by Moynihan *et al,*^29^ financial ties to drug manufacturers that were disclosed in the scientific literature were only reported in 39% of the news stories. In our analysis, disclosure was present in only 3% of articles, which may reflect the more fast-moving nature of the pandemic news coverage. In spite of potential logistical difficulties, we echo Caulfield^16^ in his demand that all “reporters should always ask for and researchers should always offer information about [financial associations]”.

There are, admittedly, limitations to disclosure. Kassirer points out that disclosure currently tells us nothing about the magnitude of CoI.^39^ In addition, the interpretation of declared CoI can be subtle, as the emphasis is on complete disclosure of any CoI that may potentially influence an author outside of any judgement of their actual influence.^40^ It is not known whether this distinction would be appreciated by those unversed in the particularities of scientific CoI declarations. Researchers may be understandably reluctant to put this to the test as news stories about scientific CoI are often high profile. In a 10-year analysis of news media coverage of scientific CoI, McCormas and Simone found that nearly 1 in 10 stories appeared on the front page, suggesting a high degree of newsworthiness.^40^ Finally, journalists themselves may have undisclosed CoI that would impede truly impartial reporting.^16^

Despite these obstacles, we would argue that undisclosed CoI degrades public confidence in medical research, to the detriment of the whole scientific community. We would recommend that these principles are extended to more settings. We call on all academics to declare any potential CoI when providing commentary to the mass media. We encourage journalists to ask for and report any CoI in their interviewees, so that readers can judge their comments in full light of the facts. As Caulfield puts it,^16^ complete transparency should now be the understood standard practice. Through these measures, the academic voice will retain its credibility in public health issues.

**What is already known on this subject**

- Considerable public funding was spent on vaccines and antiviral medication during the 2009 to 2010 A/H1N1 pandemic.
- Subsequently, there were concerns over competing interests of academics serving on scientific advisory committees during the pandemic.
- Many academics also provide media commentary on emerging health risks, and the media has been shown to influence public risk perception and demand for new drugs.

**What this study adds**

- Academics with competing interests were more likely to predict a higher risk to the public from the pandemic than official agencies compared with those without any competing interests.
- Academics promoting the use of antiviral medication were more likely to have a competing interest than those not commenting on its use.
- Given the evidence of competing interests among academics providing media commentary, these should be declared before media interviews in order for public health to retain its independent voice.
